# EHMTI-0227. Therapeutic effectiveness of sphenopalatine ganglion (SPG) stimulation for cluster headache – pathway registry study interim results at 6 months

**DOI:** 10.1186/1129-2377-15-S1-C26

**Published:** 2014-09-18

**Authors:** TP Jürgens, R Jensen, H Kaube, A Böger, C Gaul, A Goodman, A Caparso, A May

**Affiliations:** 1 Headache Clinic Dept. of Systems Neuroscience, Medical Center Hamburg-Eppendorf, Hamburg, Germany; 2 Danish Headache Center, Glostrup Hospital University of Copenhagen, Copenhagen, Denmark; 3 Neurologie und Kopfschmerzzentrum Muenchner Freiheit, Neurologie und Kopfschmerzzentrum Muenchner Freiheit, Munich, Germany; 4 Schmerzzentrum, Rotes Kreuz Krankenhaus, Kassel, Germany; 5 Migräne und Kopfschmerzklinik, Migräne und Kopfschmerzklinik, Königstein, Germany; 6 Autonomic Technologies Inc., Autonomic Technologies Inc., Redwood City California, USA

## Background

The Pathway Registry is an open label registry of SPG stimulation therapy for cluster headache treatment. In a previous randomized, double-blind, multi-center study (Pathway CH-1 study) 68% of patients experienced clinically significant improvements.

## Aim

The aim of this interim analysis is to evaluate acute and/or preventive therapeutic effectiveness at 6 months following insertion of an SPG neurostimulator.

## Method

Therapeutic effectiveness (acute pain response following SPG stimulation and/or attack frequency reduction) was analyzed during the first six months following SPG neurostimulator insertion. Acute pain responders achieved relief from moderate or greater pain, or freedom from mild pain in ≥50% of analyzable attacks (with completed diary questions). Frequency responders achieved ≥50% attack frequency reduction at the six month study visit (evaluated over the previous four weeks), versus the 4 week baseline period.

## Results

49 patients have been enrolled, 18 completed follow-up through six months[RJ1] (189 days post-insertion, range 149-238). Average baseline attack frequency was 28.4 attacks/week (range 0-70), and the average attack frequency at 6 months was 17.3 attacks/week (range 0-70), a 40% reduction. 67% (12/18) were responders. Of the 12 responders, 67% (N=8) were acute responders, treating 86% of their attacks effectively (N=546). 75% (N=9) were frequency responders; frequency reduced by 90% (from 22.3 at baseline to 2.1 (range 0-8) attacks/week). 5 patients were both acute and frequency responders.

## Conclusion

Interim data from a registry of cluster headache patients continues to demonstrate the effectiveness of SPG stimulation therapy.

